# Comparison of different treatment approaches in chronic non-bacterial osteomyelitis.

**DOI:** 10.1186/1546-0096-13-S1-P203

**Published:** 2015-09-28

**Authors:** M Kostik, I Chikova, V Masalova, M Dubko, L Snegireva, E Isupova, O Kalashnikova, V Avramenko, A Denisov, D Vorypin, D Philippov, S Peredereev, D Malamashin, A Pershin, E Malyarova, M Bakin, V Evseev, A Mushkin, V Chasnyk

**Affiliations:** 1 Saint-Petersburg State Pediatric Medical University, Hospital Pediatry, Saint Petersburg, Russian Federation; 2 Saint-Petersburg State Pediatric Medical University, Pediatric Surgery, Saint Petersburg, Russian Federation; 3 Federal State Budget Institue “Science research Institute of Phthisiopulmonology Ministry of Health RF”, Pediatric Surgery, Saint Petersburg, Russian Federation

## 

Chronic non-bacterial osteomyelitis (CNO) is a heterogenous group of immune-mediated inflammatory bone diseases, which often co-exist with other rheumatic diseases. There are no approved treatments for CNO, except non-steroid anti-inflammatory drugs (NSAID). The efficacy of methotrexate (MTX), sulfasalazine, pamidronate (PAM), anti-IL1 and TNFα-inhibitors was shown in different reports, but there are some concerns about safety of pamidronate due to long-term accumulation and persistation in bone.

The aim of our study was to compare the efficacy of non-randomized different treatment approaches in pediatric patient cohort with CNO.

## Materials

37 children (16 M and 21 F) with CNO from medical centers in Saint Petersburg. The average age at the onset of disease was 8.5 years (5.9÷10.5), the number of foci - 3.0 (2.0÷6.5, incl. multifocal cases in 78.4%), fever at the onset -37.8%, spine involvement - 32.4%, positive family autoimmune diseases (AID) history - 8.1%, concomitant AID - 64.9%. NSAID was the first-line treatment for non-vertebral cases, as well as PAM for vertebral involvement. Second-line treatment includes MTX, PAM and TNFα-inh. Dynamics of pain, patient's (PVAS) and physician's (MDVAS) assessment of CNO activity we evaluated.

## Results

According to the NSAID, MTX, PAM and TNFα-inh groups next data were registered:

PVAS: -26.2% (p=0.05), -14.6% (p=0.06), -84.7% (p=0.0002),-75.6% (p=0.012);

pain: -36.4% (p=0.028), -15.6% (p=0.31), -84.8% (p=0.0002), -82.6%, p=0.012);

MDVAS: -33.8% (p=0.08); +2.4% (p=0.24),-81.4% (p=0.0002), -75.8%, (p=0.012), respectively.

The therapy was effective in 38.9%, 57.1%, 83.3% and 88.8% respectively (log-rank test, p=0.012). TNFα-inh usually used as second-third line treatment in cases where other options, especially PAM were fall.

## Conclusions

The most effective treatment approaches for CNO were PAM and TNFα-inh. The randomized controlled trials for assessment efficacy and safety of these medications is mandatory to confirm these results.

